# Evaluating a Two-Tiered Parent Coaching Intervention for Young Autistic Children Using the Early Start Denver Model

**DOI:** 10.1007/s41252-022-00264-8

**Published:** 2022-05-30

**Authors:** Lauren E. van Noorden, Jeff Sigafoos, Hannah L. Waddington

**Affiliations:** School of Education, Victoria University of Wellington, Wellington, New Zealand

**Keywords:** Naturalistic developmental behavioral intervention, Early Start Denver Model, Parent-implemented, Autism, Early intervention

## Abstract

**Objectives:**

Early intervention can improve the outcomes of young autistic children, and parents may be well placed to deliver these interventions. The Early Start Denver Model (ESDM) is a naturalistic developmental behavioral intervention that can be implemented by parents with their own children (P-ESDM). This study evaluated a two-tiered P-ESDM intervention that used a group parent coaching program, and a 1:1 parent coaching program. We evaluated changes in parent use of the ESDM and parent stress, as well as child engagement, communication, and imitation.

**Methods:**

Seven autistic or probably autistic children (< 60 months old) and their parents participated. A multiple-baseline design was used to compare individual changes between Baseline 1, Group Coaching (Tier 1), Baseline 2, and 1:1 Coaching (Tier 2). Parent and child behaviors were analyzed from weekly videos and graphed. Parenting stress was measured.

**Results:**

All parents improved in their use of ESDM strategies after the Tier 1 intervention. Changes in parent fidelity during Tier 2 were mixed, but all parents maintained higher than baseline levels of fidelity. Six parents demonstrated above 75% ESDM fidelity in at least one session. There were positive changes in parent stress levels pre- post-intervention. Positive results were found for most children’s levels of engagement, imitation, and communication. There were significant positive relationships between parent fidelity and both child engagement and child functional utterances.

**Conclusions:**

Group P-ESDM is a promising approach for improving parent fidelity and some child outcomes. Future randomized and controlled studies of group P-ESDM, using standardized outcome measures, are warranted.

**Supplementary Information:**

The online version contains supplementary material available at 10.1007/s41252-022-00264-8.

Autism is a neurodevelopmental condition characterized by challenges in social communication and social interaction as well as the presence of restricted, repetitive patterns of behavior, interests, or activities (American Psychiatric Association, 2013). It is now estimated that 1 in 59 children are autistic (Baio et al., 2018). Early intervention (EI) can support the development of autistic children (Dawson & Bernier, 2013; Debodinance et al., 2017) and can lead to improvements in areas such as communication, play skills, cognition, and social engagement (Tiede & Walton, 2019). Naturalistic developmental behavioral interventions (NDBIs) are a particularly promising EI approach with a strong evidence base (Sandbank et al., 2020; Schreibman et al., 2015; Tiede & Walton, 2019). NDBIs are characterized by several program features, commonly (a) pivotal or developmentally significant learning targets, such as teaching joint attention and imitation skills, (b) a strong focus on building positive social relationships, (c) using behavioral teaching strategies within naturally motivating and ecologically valid activities, and (d) ensuring children experience high levels of success (Schreibman et al., 2015).

Research and practice guidelines have established that parent involvement is essential for EI (Ministries of Health & Education, 2016; National Institute for Health & Care Excellence, 2013; National Research Council, 2001; Wallace & Rogers, 2010). Parent-mediated interventions (PMIs), including parent-mediated NDBIs, are also a promising approach for supporting the learning and development of autistic children (Nevill et al., 2018; Oono et al., 2013; Wong et al., 2015). PMIs are associated with improvements in many child outcomes including social skills, communication, and cognition (Nevill et al., 2018; Oono et al., 2013). There may also be benefits for parents who participate in PMIs including improving parental stress (McConachie & Diggle, 2007; Weitlauf et al., 2020) and self-efficacy (Brian et al., 2017; Noyan Erbaş et al., 2020), as well as parent sense of competence and family cohesion (Koegel et al., 2002; Nevill et al., 2018).

The rationale for PMIs includes the possibility that parents are well-placed to deliver intervention throughout their children’s everyday routines, which may lead to greater consistency of intervention and thus improved child outcomes (Nevill et al., 2018; Oono et al., 2013). PMIs can also be a time and cost-effective alternative to intensive clinician-implemented interventions (Shalev et al., 2020). This suggests the value of providing coaching to parents in relation to providing PMIs, especially where there is a lack of funding for direct interventionist support and/or shortages of trained service providers to deliver interventions for young children (Abouzeid et al., 2020).

The Early Start Denver Model (ESDM) is a manualized NDBI for children aged 12–48 months who are, or are suspected to be, autistic (Rogers & Dawson, 2010). The ESDM can have positive outcomes when delivered by parents (Baril & Humphreys, 2017; Fuller et al., 2020; Ryberg, 2015; Waddington et al., 2016). The ESDM combines relationship-focused developmental interventions, with the principles of applied behavior analysis. It aims to provide naturalistic learning opportunities that optimize child motivation and create affectively rich socio-communication exchanges to target skill development. Skills across several domains are targeted, including receptive and expressive communication, social skills, cognition, imitation, joint attention, play, fine and gross motor, and personal independence (Rogers & Dawson, 2010). The ESDM has been effectively implemented by teachers, clinicians, and parents after receiving coaching to ensure fidelity of implementation (Baril & Humphreys, 2017; Fuller et al., 2020; Ryberg, 2015; Waddington et al., 2016).

Several different approaches have been employed for coaching parents to implement ESDM. Most such parent-implemented ESDM (P-ESDM) studies have used a 1:1 coaching approach, where a coach works directly with the parent to support their use of the ESDM techniques. Parent coaching has occurred in research clinics (Rogers et al., 2012b; Weitlauf et al., 2020), in the parents’ home (Waddington et al., 2019), or in a mixture of clinic and home settings (Rogers et al., 2019). Parent coaching has also been conducted via telehealth (Vismara et al., 2012, 2013, 2016), or through community service providers (Mirenda et al., 2022; Rogers et al., 2022). In addition, Abouzeid et al. (2020) used a mixed approach with one initial group parent coaching workshop, followed by individual parent coaching sessions. Zhou et al. (2018) used a hybrid coaching approach where trainee ESDM therapists modeled the ESDM strategies for the first 12 weeks, and parents only worked directly with their own children in the last 12 weeks.

Most P-ESDM studies have evaluated changes in parent fidelity of implementation using the ESDM teaching fidelity rating system (ESDM fidelity scale; Rogers & Dawson, 2010). The impact of P-ESDM on parent fidelity differs between studies. Vismara et al. (2012), for example, found that all parents in their study who received coaching on the use of the P-ESDM reached 80% fidelity. Other studies have found that parents did not reach 80% fidelity overall (e.g., Rogers et al., 2022; Waddington et al., 2019), or some but not all parents reached the 80% fidelity criterion (e.g., Vismara et al., 2016). Mirenda et al. (2022) reported positive impacts from coaching on other aspects of parent behavior, such as scaffolding and following the child’s lead. In addition to showing that parents can learn to implement ESDM techniques with fidelity, several studies have also found that participating in P-ESDM can positively impact parent stress levels (Estes et al., 2014; Rogers et al., 2012b; Weitlauf et al., 2020; Zhou et al., 2018).

The impact of P-ESDM intervention on child outcomes differs between studies. Some studies have reported improvements in a range of targeted child behaviors (e.g., Vismara et al., 2009, 2012, 2013; Mirenda et al., 2022; Waddington et al., 2021; Zhou et al., 2018), while other studies have shown no intervention effect compared to the control group (Rogers et al., 2012a, b, 2022; Vismara et al., 2016). This latter finding is not inconsistent with the larger literature on PMIs, in which several studies have reported small to moderate effect sizes on some child outcomes (Nevill et al., 2018; Oono et al., 2013).

To improve outcomes for children participating in P-ESDM, it may be helpful to provide a longer period of intervention, beyond the standard 12-week duration (Abouzeid et al., 2020; Rogers et al., 2012b). Along these lines, three studies have evaluated the effect of longer P-ESDM interventions (Mirenda et al., 2022; Rogers et al., 2022; Zhou et al., 2018). However, Rogers et al. (2022) and Zhou et al. (2018) did not involve many hours of PMI. Mirenda et al. (2022) ran a 24-week program with one 60-min coaching session per week, based on feedback from a pilot study that found two 60-min parent coaching sessions per week for 12 weeks was unachievable for participating families. The results showed that children in the P-ESDM condition made significantly greater gains in their receptive language skills than children in the community condition (Mirenda et al., 2022).

Group delivery of P-ESDM may be one way to increase the accessibility and sustainability of the approach, as it could be seen as a more practical and feasible approach than delivery based on therapist implemented, or 1:1 parent coaching therapy sessions. Additionally, group parent coaching may help to facilitate social learning and decrease the stigma that may be associated with a diagnosis of autism spectrum disorder (ASD; Sengupta et al., 2020). Group delivery of parent coaching has been found to be feasible and effective in other NDBIs (Hardan et al., 2015; Sengupta et al., 2020), but to date, no research has evaluated the effectiveness of group coaching in the P-ESDM. Innovative methods of intervention delivery that are sustainable in community programs are needed in the NDBI field (Schreibman et al., 2015); therefore, evaluating group delivery of P-ESDM seems warranted.

Given the variable parent and child responses to intervention both within and between parent coaching studies, there is a need for parent education approaches that are flexible to meet family needs and can be adapted based on parent response to treatment (Phaneuf & McIntyre, 2011). A stepped-care approach is a multi-tiered program with less intensive supports provided initially, and more intensive supports provided as needed (Bower & Gilbody, 2005). This approach to autism service delivery might improve outcomes for children and their families through individualizing supports, while perhaps also promoting a more efficient prioritization of resources (Green, 2019; Steever, 2011; Webb et al., 2014). In a stepped-care approach to parent coaching, initial low-intensity programs — such as self-guided reading or completion of online modules, or group parent coaching programs — might be used. For families who still need more support, slightly more intensive programs could include 1–1 parent coaching in person, or via telehealth. For parents who do not respond to these earlier low-intensity tiers of intervention, additional more intensive supports that are adapted to the needs of the family may need to be offered. Multi-tiered parent coaching programs may be a way to overcome treatment barriers, optimize intervention efficiency, and improve the effectiveness of parent-mediated interventions (McIntyre & Phaneuf, 2008; Phaneuf & McIntyre, 2011; Wainer et al., 2021).

This single-case research evaluated the effectiveness of a two-tiered ESDM parent coaching intervention for improving parent use of the ESDM strategies, and for improving imitation, joint engagement, and communication for autistic children. The two tiers of intervention consisted of a 10-week group P-ESDM coaching intervention (Tier 1) and a 10-week 1:1 P-ESDM intervention for parents who do not reach fidelity in Tier 1 (Tier 2). Specifically, this research sought to answer (1) whether 1.5 h per week of group ESDM parent coaching for 10 weeks increases parent use of the ESDM intervention procedures to 80% fidelity, (2) for parents who do not reach fidelity after participating in the group ESDM parent coaching, does 1 h per week of 1:1 ESDM parent coaching for 10 weeks increase parent use of the ESDM intervention procedures to 80% fidelity, and (3) whether parent participation in both a 10-week group coaching intervention and a 10-week 1:1 parent coaching intervention produces an increase in imitation, expressive language, and joint engagement for young autistic children.

## Method

### Participants

Seven parents and their autistic children were recruited for this research through a university-based autism clinic. Participants were recruited in two groups. Four families participated in Group 1 and three families participated in Group 2. Parents were eligible to participate if they (a) were a caregiver for, and lived in the same household as, the participating autistic child and (b) had not received any P-ESDM previously. The demographic characteristics of the participating parents, listed under pseudonyms, are summarized in Table 1.

**Table 1:** Family demographic characteristics

Parent pseudonym	Group 1				Group 2		
	Heather	Holly	Kelly	Kiran	Amanda	Merry	Sam
C*hild pseudonym*	*Zack*	*Charles*	*Elijah*	*Muhammed*	*Steve*	*Harry*	*Dominic*
Primary participant	Mother	Mother	Mother	Mother	Mother	Father	Father
Additional participants (sessions attended)	–	Father (2) Grandma (1) Grandad (1)	–	Father (2)	Father (1)	Mother (20)	Mother (13)
Marital Status	De facto*	Single	Married	Married	Married	De facto	Married
Number of people living in the home	Three (mother, sibling, child)	Seven (maternal great-grandparents, grandparents, mother, child)	Four (mother, father, sibling, child)	Four (mother, father, sibling, child)	Three (mother, father, child)	Four (mother, father, sibling, child)	Nine (maternal grandparents, mother, father, 4 siblings, child)
Employment	Part-time	Part-time	Part-time	Stay at home	Full-time	Full-time	Full-time
Education	High School	High school	Bachelor’s	Master’s	Doctorate	Trade certified	Trade certified
Language(s) spoken at home	English	English	Māori, English	Urdu	English, Cantonese	English	Samoan, English

*Zack’s parents were living separately due to international COVID-19 restrictions throughout this research. *Child*, child participating in this research

Parents also completed the Broad Autism Phenotype Questionnaire (BAPQ; Hurley et al., 2007) which measures autism-like characteristics including (a) aloof personality, (b) rigid personality, and (c) pragmatic language using a 36-item Likert-type survey. The BAPQ has high specificity and sensitivity (Hurley et al., 2007), and has good internal consistency (Sasson et al., 2013). Normative cut-off scores (the score value which indicates the presence of an autism-like phenotype) with high specificity were used (Sasson et al., 2013). The results are summarized in Table 2.

**Table 2:** Parent BAPQ average item score results

BAPQ items	Group 1				Group 2			Cut-off scores*	
	Heather	Holly	Kelly	Kiran	Amanda	Merry	Sam	(Women)	(Men)
Aloof personality	3.00	2.83	1.92	3.17	2.08	3.50	4.00	*3.45*	*4.13*
Rigid Personality	2.33	2.33	2.17	2.25	2.08	2.75	*4.58*	*2.94*	*3.23*
Pragmatic Language	*3.92*	2.83	2.08	2.92	2.25	1.08	*4.00*	*3.17*	*3.91*
Total Score	3.08	2.67	2.06	2.78	2.14	2.44	*4.19*	*3.17*	*3.55*

Cells with values in italics indicate scores above the cut-off and therefore suggest a phenotypic expression of characteristics similar to ASD. Cut-off scores are taken from Sasson et al. (2013)

The child participants were eligible if they (a) were between 12 and 54 months old, (b) had an independent diagnosis of ASD or had a high likelihood of ASD based on the Childhood Autism Rating Scale, Second Edition (CARS-2; Schopler et al., 2010), (c) could walk independently, (d) were not receiving more than 10 h of early intervention services per week, (e) had not received more than 25 h of ESDM therapy in the past 12 months, and (f) were not receiving any additional ESDM therapy while participating in this research. The participating child demographic information, CARS-2 (Schopler et al., 2010), and Vineland Adaptive Behavior Scales, Third Edition (Vineland-3; Sparrow et al., 2016) results are summarized in Table 3.

**Table 3:** Child demographic characteristics, Vineland-3 results, and CARS-2 results

Variables	Charles	Dominic	Elijah	Harry	Muhammed	Steve	Zack
Gender	Male	Male	Male	Male	Male	Male	Male
Ethnicity	NZ European	Samoan	Māori— NZ European	Māori —NZ European	Pakistani	Chinese —NZ European	NZ European
Age at start of study (years:months)	3:3	3:8	4:4	4:4	3:1	3:0	3:0
Age at diagnosis (ASD)	2:6	4:1	2:11	4:4	2:9	3:5	2:0
Verbal language	Single words	Short phrases	Short phrases	Short phrases	Non-verbal	Phrase speech	Minimally verbal
Hours of other services received per week (including ECE)							
CARS-2 (score) *Signs of ASD*	(27.5) *Minimal*	(30.5) *Mild-to-moderate*	(30.5) *Mild-to-moderate*	(30) *Mild-to-moderate*	(42.5) *Severe*	(34.5) *Mild-to-moderate*	(38) *Severe*
Vineland-3							
Adaptive behavior composite	76	60	71	68	66	82	62
Communication (standard score)	75	73	65	67	59	83	49
Daily living (standard score)	84	78	73	73	73	92	74
Socialization (standard score)	76	19	76	64	64	79	56
Motor skills (standard score)	89	71	71	74	81	85	74

*NZ*, New Zealand. Verbal language ability was defined as: non-verbal (no functional words or word approximations); minimally verbal (uses < 10 functional words/word approximations); single words (uses > 10 functional words/word approximations); short phrase speech (sometimes uses 2–3 word phrases); phrase speech (typically uses 3 + word phrases), fluent (no language delay compared to same-age peers). Vineland-3 scores each have a normative mean of 100, and a normative standard deviation of 15

### Procedures

#### Setting and Personnel

All baseline sessions, group coaching sessions, and 1:1 parent coaching sessions took place at a university-based clinic in New Zealand. The first author was the parent coach for all sessions. The coach was a certified ESDM therapist who had also completed the ESDM parent coaching training. She had 5 years of experience working with autistic children and was a doctoral student.

#### Research Design

A non-concurrent multiple baseline across groups design was used for this research (Harvey et al., 2004). This design allowed for experimental control, while also providing the flexibility to work with the logistical constraints of conducting research in an educational context, and the inability to reverse any intervention effects. Within each group, an AB^1^AB^2^ design was used to evaluate individual participant outcomes, with A being baseline conditions, B^1^ the group coaching intervention (Tier 1), and B^2^ being the 1:1 coaching intervention (Tier 2). Reinstatement of baseline conditions after B^1^ created opportunities to probe for maintenance after this phase of intervention. A 3-week COVID-19 lockdown occurred mid-way through the intervention. No data was collected during the lockdown, and thus this served as an unintended maintenance probe. The two groups of participants moved non-concurrently through the following sequential phases: (a) pre-baseline, (b) baseline, (c) group coaching intervention (Tier 1), (d) post-intervention (Tier 1), (e) reinstated baseline, (f) 1:1 coaching intervention (Tier 2), (g) post-intervention (Tier 2).

#### Phases

##### Pre-baseline

The first author met individually with each family to obtain informed consent and to conduct the pre-assessments including the demographic survey, the BAPQ (Hurley et al., 2007), the Parenting Stress Index – Short Form (PSI; Abidin, 2012), and the Vineland-3 (Sparrow et al., 2016). The researcher also conducted a CARS-2 (Schopler et al., 2010), and an ESDM curriculum checklist (Rogers & Dawson, 2010) with each child, in the clinic. The ESDM curriculum checklist is a goal-setting tool that includes skills from a range of developmental domains, split over four developmental age periods; level one (12–18 months), level two (18–24 months), level three (24–36 months), and level four (36–48 months) (Rogers & Dawson, 2010). The curriculum checklist is administered through play-based assesment and parent report.

##### Baseline

Groups of parents were randomly allocated to different baseline phases of either three or five probes, with additional probes implemented as needed until a stable baseline with no increasing trend in parent fidelity was established for each parent–child dyad (see Dependent Variables for a definition of fidelity). Group allocation to baseline length was necessary to allow all parents in a group to begin the intervention at the same time (Harvey et al., 2004). Baseline probes were 10-min videos in the clinic. Parents were asked to play with their child as they normally would, and the researcher did not interact with the parent or child during the probe.

##### Group Coaching Intervention (Tier 1)

During the group parent coaching intervention phase, parents met with the researcher at the clinic, in groups, for 60–90-min sessions, once per week, for 9–10 weeks. The program was intended to be 10 weeks; however, due to a COVID-19 lockdown, Group 2 only received 9 weeks of group intervention. Parents attended sessions with their child, and other parents/caregivers and siblings were also welcome to attend. The P-ESDM teaching content was drawn from the Help is in Your Hands website (Rogers & Stahmer, 2022) and the Parent ESDM Manual (Rogers et al., 2012a). Parents were given handouts of the content and were encouraged to watch the Help is in Your Hands modules. Content included topics such as (a) capturing the child’s attention, (b) structuring joint activity routines, (c) using behavioral strategies to teach new skills, and (c) teaching communication objectives. During coaching discussions, parents sat with the parent coach at a table at one end of a large room, while student volunteers minded the children at the other end of the same room. Coaching discussions involved parents reflecting on their goals and homework from the previous week, followed by a group discussion of a new ESDM topic, with collaborative problem solving, and time spent relating the content to each child’s goals. Then, at least 10-min of each session was dedicated to parents practicing the strategies with their own child with feedback from the parent coach. See supplementary materials Table 1 for the Group Coaching procedural integrity checklist. Each week, either before or after the group coaching session, a 10-min video sample was recorded of each parent–child dyad playing and interacting together in a clinic therapy room. The researcher was present but did not coach or interact with the parent or child while the video was being recorded.

##### Post-intervention (Tier 1)

An independent researcher conducted semi-structured interviews. The qualitative results from the interviews will be published separately.

##### Reinstated Baseline

Baseline was reinstated the week after group coaching finished for Group 1, and 3 weeks of post-group coaching for Group 2, after the COVID-19 lockdown. Baseline procedures during this phase were the same as the procedures for the first baseline.

##### 1:1 Coaching Intervention (Tier 2)

Tier 2 was offered to all parents from Groups 1 and 2. All parents chose to participate in the Tier 2 individualized parent coaching. The first 1:1 session with each family was a review of the child’s goals and progress based on the ESDM curriculum checklist (Rogers & Dawson, 2010), and new goals were set for each child as needed. Children did not have to attend this first session. The remaining coaching sessions were 50-min, individual coaching sessions with both the parent and child, in the clinic, for nine sessions over a maximum of 11 weeks. Additional parents and caregivers were welcome to attend, but any additional siblings were encouraged to remain outside of the therapy room for the duration of the session. After greeting the family and collecting parent-log data from the previous week, the researcher took a 10-min video of the parent playing with their child uninterrupted. Following the video, parents were invited to reflect on their use of the ESDM strategies. The focus for each session was then decided on collaboratively from any of the areas in which the parents were not yet using ESDM strategies at fidelity (scores of 4–5 on the ESDM fidelity scale). The remainder of the session involved discussion, guided practice, reflection, then goal setting in line with the P-ESDM Coaching Fidelity Rating Tool (Rogers et al., 2021).

##### Post-intervention (Tier 2)

Post-intervention assessments were conducted after the 1:1 parent coaching intervention finished. The PSI (Abidin, 2012) was re-administered to parent participants to evaluate any changes in parental stress. An independent researcher also conducted semi-structured interviews, which again will be published separately.

### Measures

#### Parent Log of Estimated Intervention Hours

Parents were asked to estimate the total time spent using the ESDM strategies each day per week and record it on a weekly log.

#### Procedural Integrity

Procedural integrity was measured in 20% of sessions in each phase (Baselines, Tier 1 coaching, and Tier 2 coaching). A research assistant live coded the procedural integrity of entire sessions in both Tiers 1 and 2. In Tier 1 a 12-item checklist was used (see supplementary materials Table 1). Items on the checklist related to the structure and duration of the session, content, and coaching activities, as well as reflection and goal setting. In Tier 2, the P-ESDM Coaching Fidelity Rating Tool (Rogers et al., 2021) was used to rate the coach’s fidelity. This rating tool is a 14-item Likert-type scale. Items on the scale relate to both the structure of the session, and the characteristics of the coaching approach (e.g., a collaborative approach, reflective practices, non-judgmental approaches). In both baseline phases, procedural integrity was measured from the video recordings using a 5-item checklist written by the researcher, with items such as “video lasted for 10-min” (see supplementary materials Table 2).

#### Dependent Variables

##### Parent Fidelity

The primary parent dependent variable was the fidelity of implementation as measured by the ESDM fidelity scale (Rogers & Dawson, 2010). The fidelity scale is divided into 13 items. Each item can be rated on a Likert scale from 1 to 5 (reflecting poor to consistent use of each technique). Scores of four and five on the fidelity scale demonstrate that the parent was using that strategy “usually” and “consistently” respectively. The percentage of the 13 items that the parents were using “usually or consistently” was reported for each session, using the calculation: (Number of techniques used “usually” or “consistently”)/(total number of techniques (13)) × 100 = % fidelity score.

This aligns with previous P-ESDM research (Waddington et al., 2019) and shows greater sensitivity to changes in parent use of ESDM strategies than deriving a percentage from the sum of the 13 individual item scores.

##### Parenting Stress

The Parenting Stress Index-Short Form (PSI) was used to evaluate levels of parental stress (Abidin, 2012). The PSI is a 36-item self-report measure, with three sub-scales of (a) parental distress, (b) parent–child dysfunctional interaction, and (c) difficult child (Abidin, 2012). It has good validity, internal consistency, and test–retest reliability (Abidin, 2012).

##### Child Outcomes

The child-dependent variables (DVs) were specific child behaviors during parent–child interactions: joint engagement, imitation, and measures of communication. These measures are defined operationally in supplementary Table 3. To measure child outcomes, each 10-min video was divided into 60, 10-s intervals. Whole interval recording (Kennedy, 2005) was used to measure whether the child was engaged with the parent for the entire 10-s interval, and partial interval recording (Kennedy, 2005) was used to measure whether specific imitation and communication behaviors occurred within each interval. Both the whole interval and partial interval data are reported as percentages, calculated using the formula: (Number of intervals containing DV)/(Total number of intervals) × 100 = % occurrence of DV.

#### Interobserver Agreement

The first author coded all videos. To calculate inter-observer agreement (IOA), an independent observer, who was blind as to which phase of intervention the participants were in, coded all DVs on 20% of the videos from each phase. The observer had an undergraduate degree in psychology, but no formal training in the ESDM or other autism interventions. The lead researcher trained the independent observer to use the DV coding systems using practice videos until 80% agreement was obtained. IOA on child DVs was calculated using interval agreement (Kennedy, 2005) where every interval that both the researcher and observer coded the same occurrence or absence of a behavior counted as an agreement. IOA percentage was calculated using the formula: (number of agreements)/(total number of intervals) × 100 = % agreement.

IOA for parent use of strategies was calculated using adjacent agreement on the 5-point ESDM fidelity Likert scale. That is, if the researcher and observer coded one of the 18 fidelity items the same (1–5) or within one point of each other, it was counted as agreement. To ensure transparency, exact agreement rates are also reported (in brackets).

### Data Analyses

Visual analysis and descriptive statistics were used to evaluate the parent fidelity and child data from graphs across each phase. Visual analysis can be used to infer a causal relationship between the independent variable and any changes in the dependent variables in single-case research designs (Kratochwill et al., 2010). Additionally, an effect size for each variable was calculated using Tau-U (Parker & Hagan-Burke, 2007; Vannest & Ninci, 2015). The Tau-U is an appropriate method for calculating effect sizes with small data sets in single-case research designs as it includes both trend and level, controls for positive trends in baseline, discriminates well at the upper and lower limits, and is distribution free (Parker et al., 2011; Vannest & Ninci, 2015). The Tau-U was calculated using the matrix provided at http://www.singlecaseresearch.org/calculators/tau-u. Effect sizes are reported as small (≤ 0.20), moderate (0.20–0.60), large (0.60–0.80) or very large (≥ 0.80) (Vannest & Ninci, 2015). Spearman’s rank correlations were used to evaluate any relationship between changes in parent fidelity and changes in child outcomes. Values between ± 0.1 and ± 03 reflect weak relationships, values between ± 0.3 and ± 0.5 reflect moderate relationships, and values between ± 0.5 and ± 1 indicate a strong relationship (Xiao et al., 2016).

## Results

### Attrition

All families completed the intervention, except Kelly who withdrew halfway through Tier 2 (following a 3-week COVID lockdown) due to increased personal health, family, and work demands.

### Parent Fidelity

**Fig. 1:**
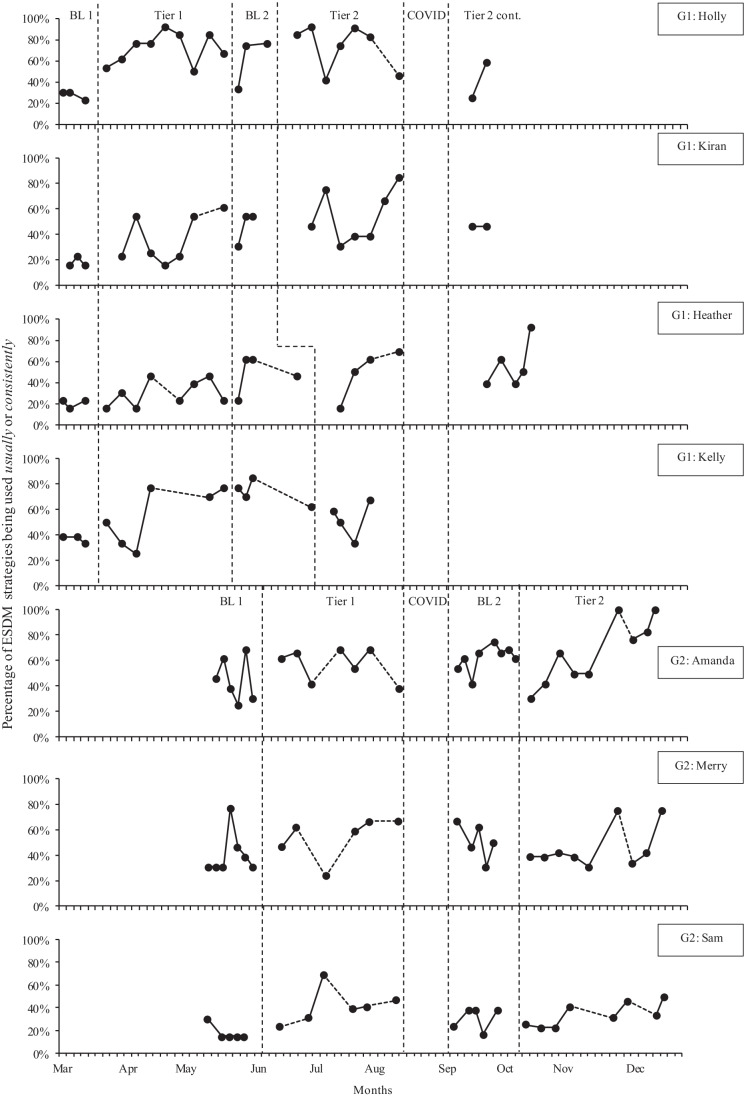
Percentage of ESDM strategies implemented at fidelity across individual parents and study phases. Note: BL, Baseline; COVID, COVID lockdown; Tier 2 cont., Tier 2 continued after the COVID lockdown. G1, Group 1; G2, Group 2. A dashed line between data points indicates a gap of more than 1 week

Figure 1 shows the percentage of ESDM strategies being used usually or consistently (i.e., “at fidelity”) for all parents across phases. Table 4 shows changes in parent fidelity across phases, by mean, standard deviation, percentage of sessions with above 75% fidelity, and Tau-U effect sizes.

**Table 4:** Parent fidelity: means, standard deviations, percentages of sessions above 75% fidelity, and Tau-U values across all parents and all phases

	Parent	Baseline 1		Tier 1			Baseline 2				Tier 2			
		Mean (SD)	Sessions ≥ 75% (≥ 80%) fidelity	Mean	Sessions ≥ 75% (≥ 80%) fidelity	Tau-U (BL1/Tier 1)	Mean	Sessions ≥ 75% (≥ 80%) fidelity	Tau-U (BL1/BL2)	Tau-U (Tier 1/BL2)	Mean	Sessions ≥ 75% (≥ 80%)fidelity	Tau-U (BL1/Tier 2)	Tau-U (BL2/Tier 2)
1	Holly	28% (4.44)	0 (0)	72% (14.71)	5 (3)	***1.00***	62% (24.63)	2 (0)	***1.00***	* − 0.33*	66% (24.50)	5 (4)	***0.85***	0.22
	Kiran	18% (4.44)	0 (0)	37% (19.01)	0(0)	**0.71**	46% (13.32)	0 (0)	***1.00***	0.33	52% (18.46)	2 (1)	***1.00***	0.07
	Heather	21% (4.44)	0 (0)	26% (12.63)	0 (0)	0.42	48% (18.18)	0 (0)	***0.83***	**0.63**	53% (21.82)	1 (1)	***0.81***	0.11
	Kelly	37% (2.96)	0 (0)	55% (22.62)	2 (0)	0.39	73% (7.96)	2 (1)	***1.00***	**0.61**	52% (14.23)	0 (0)	0.58	* − 0.88*
2	Amanda	45% (17.36)	0 (0)	57% (12.92)	0 (0)	0.43	62% (10.32)	1 (0)	0.56	0.18	67% (25.19)	4 (3)	0.54	0.10
	Merry	41% (17.04)	1 (0)	54% (16.82)	0 (0)	0.40	51% (14.06)	0 (0)	0.46	* − 0.13*	46% (16.89)	2 (0)	0.32	* − 0.27*
	Sam	18% (6.88)	0 (0)	42% (15.84)	0 (0)	***0.90***	31% (10.43)	0 (0)	***0.84***	* − 0.47*	34% (10.64)	0 (0)	***0.83***	0.20

*1*, Group 1; *2*, Group 2. Bold indicates large effect sizes (> 0.50), bold-italics indicates very large effect sizes (> 0.80), italics indicates a negative effect size (< 0)

During Baseline 1, there were stable or decreasing baseline trends across all parents, with high variability for Amanda and Merry, and low variability for the remaining parents. There was an immediate positive effect of the group coaching intervention (Tier 1) for Holly and Kelly, a flat trend for Amanda, and gradual increasing trends for all other parents, with high variability across all parents. All parents increased their mean fidelity during Tier 1 to above Baseline 1 levels. There were moderate (Heather, Kelly, Amanda, and Merry), large (Kiran), or very large (Holly and Sam) positive effect sizes across all parents. Mean fidelity then remained above Baseline 1 levels for all parents throughout all other phases of intervention.

Between Tier 1 and Baseline 2, changes in mean fidelity were mixed with some parents continuing to increase their mean fidelity with small (Amanda), moderate (Kiran), and large (Heather and Kelly) positive effect sizes. While other parents showed decreases in mean fidelity with small (Merry), to moderate (Holly and Sam) negative effect sizes. There continued to be moderate-to-high variability in parent fidelity during Baseline 2. Holly, Kiran, and Heather had increasing trends in Baseline 2 (but below Tier 1 levels), and Kelly and Merry had decreasing trends.

With the introduction of individual coaching (Tier 2), Heather, Kelly, Amanda, Merry, and Sam showed initial decreases in fidelity to below Baseline 2 mean levels. The pattern of high variability continued in Tier 2 across most parents. For group 1 parents, fidelity decreased immediately following the COVID lockdown period, and for Kiran and Holly, it did not return to pre-COVID levels. Kelly had a stable trend in Tier 2. Heather, Amanda, Merry, and Sam had increasing trends across Tier 2. Overall, five parents had higher mean fidelity in Tier 2 than in Baseline 2 and small (Kiran, Heather, and Amanda) to moderate (Holly and Sam) positive effect sizes. Mean fidelity decreased between Tier 2 and Baseline 2 for two parents, with moderate (Merry), to large (Kelly) negative effect sizes.

In total, six out of seven parents had at least one session with fidelity above 75%, and five of those parents had at least one session above 80% fidelity. In Baseline 1, only one parent (Merry) had a session above 75%. In Tier 1, two parents (Holly and Kelly) had sessions above 75% fidelity. By Baseline 2, three parents (Holly, Kelly, and Amanda) had sessions above 75%. In Tier 2, five parents (Holly, Kiran, Heather, Amanda, and Merry) had at least one session above 75% fidelity. Sam did not have any sessions above 75% fidelity across any phases.

### Parenting Stress

Table 5 shows parents’ scores on the PSI (Abidin, 2012) pre- and post-intervention. Pre-intervention data from all parents is included, as well as Harry’s mother, Jay, who was not a primary participant in this research but asked to complete this measure. Post-intervention, five out of seven parent participants returned this measure, as well as Jay. Data from pre-intervention shows that Heather, Amanda, Jay, and Sam were all showing high or clinically significant levels of stress on at least one measure. Post-intervention, no parents were showing high or clinically significant levels of stress. Five out of six parents who returned the forms showed reductions in total stress levels, ranging from a − 9 to a − 43-point difference pre- to post-intervention. Merry was the only parent who showed an overall increase in total stress with a + 2-point difference.

**Table 5:** Parenting stress index results pre- to post-intervention

	Parent	Pre-intervention				Post-intervention			
		Parental distress	Parent–child dysfunctional interaction	Difficult child	Total stress	Parental distress (score change)	Parent–child dysfunctional interaction (score change)	Difficult child (score change)	Total stress (score change)
1	Holly	22	23	29	74	22 (0)	19 **(− 4)**	24 **(− 5)**	65 **(− 9)**
	Heather	*47*	*37*	*43*	*127*	–	–	–	–
	Kelly	22	28	32	82	–	–	–	–
	Kiran	24	31	27	82	27 (+ 3)	27 **(− 4)**	23 **(− 12)**	81 **(− 18)**
2	Amanda	29	*35*	35	99	30 (+ 1)	28 **(− 7)**	23 **(− 12)**	81 **(− 18)**
	Merry	18	24	26	68	19 (+ 1)	25 (+ 1)	26 (0)	70 (+ 2)
	*Jay*	*50*	*39*	27	*119*	21 **(− 29)**	25 **(− 14)**	30 (+ 3)	76 **(− 43)**
	Sam	20	*34*	*46*	100	18 **(− 2)**	28 **(− 6)**	21 **(− 25)**	67 **(− 33)**

*1*, group 1; *2*, group 2. Heather and Kelly did not return this measure post-intervention. Cells with values in italics indicate a high or clinically significant level of stress. Bold numbers indicate a reduction in stress levels on that measure between pre- and post-intervention

### Child Outcomes

Table 6 shows the mean percentages, standard deviation, and Tau-U effect sizes for the dependent variables of engagement, functional utterances, and both vocal and gestural imitation for all child participants across all phases. The definitions and measurement of these variables are outlined in the “Dependent Variables” section. Each variable is discussed separately below.

**Table 6:** Child outcomes: Mean, standard deviation, and Tau-U values for each dependent variable across all children and phases

	Baseline 1	Tier 1			Baseline 2			Tier 2	
Outcome/children	Mean (SD)	Mean (SD)	Tau-U (BL/Tier 1)	Mean (SD)	Tau-U (BL1/BL2)	Tau-U (Tier 1/BL2)	Mean (SD)	Tau-U (BL 1/Tier 2)	Tau-U (BL2/Tier 2)
Engagement									
Charles	32% (15.12)	50% (12.74)	**0.63**	43% (10.05)	0.56	* − 0.33*	50% (16.24)	**0.63**	*0.15
Muhammed	38% (2.55)	37% (11.83)	*0.00	36% (6.31)	* − 0.44	0.05	34% (12.43)	* − 0.56	* − 0.37
Zack	23% (16.91)	20% (6.14)	* − 0.25	20% (6.83)	* − 0.25	0.03	35% (17.40)	*0.1111	*0.69
Elijah	26% (3.47)	30% (19.39)	0.17	42% (10.83)	**0.75**	0.29	26% (9.08)	0.08	* − 0.75*
Steve	39% (19.31)	63% (14.48)	**0.60**	54% (10.41)	0.35	* − 0.38*	58% (14.85)	0.56	0.15
Harry	28% (12.66)	54% (17.86)	***0.86***	39% (8.20)	0.54	* − 0.47*	30% (13.44)	* − 0.06*	* − 0.53*
Dominic	24% (14.27)	43% (24.58)	0.53	32% (12.19)	0.52	* − 0.23*	29% (11.16)	0.23	*0.15
Functional utterances									
Charles	36% (23.35)	45% (9.32)	0.26	43% (8.55)	0.11	* − 0.15*	60% (7.90)	0.56	***0.96***
Zack	12% (10.14)	13% (6.65)	* − 0.21	14% (9.61)	* − 0.25	0.06	25% (9.55)	*0.48	*0.56
Elijah	16% (0.96)	25% (9.25)	**0.72**	35% (8.86)	***1.00***	0.54	30% (9.53)	***1.00***	* − 0.25*
Steve	59% (14.63)	73% (5.52)	**0.71**	74% (5.11)	**0.75**	0.20	70% (9.08)	0.41	* − 0.32*
Harry	35% (12.65)	51% (18.61)	0.55	34% (13.79)	* − 0.03*	* − 0.53*	35% (9.44)	0.10	* − 0.02*
Dominic	0% (0.75)	38% (26.89)	***0.98***	0% (0.00)	* − 0.33*	* − 1.00*	3% (4.43)	**0.61**	**0.77**
Intentional vocalizations									
Muhammed	3% (1.67)	2% (2.03)	* − 0.38*	2% (0.00)	* − 0.67*	0.00	7% (5.40)	0.41	**0.67**
Vocal imitation									
Charles	11% (5.85)	14% (4.48)	*0.41	12% (6.67)	*0.11	* − 0.30*	11% (5.64)	*0.11	* − 0.03
Muhammed	1% (0.96)	2% (1.95)	0.33	0% (0.00)	* − 0.33*	* − 0.57*	0% (0.00)	* − 0.33*	0.00
Zack	2% (0.96)	3% (3.75)	0.04	3% (1.02)	0.25	0.09	6% (4.62)	0.52	0.42
Elijah	18% (2.55)	30% (9.49)	***0.89***	26% (2.15)	***1.00***	* − 0.29*	16% (8.96)	* − 0.17*	* − 0.69*
Steve	3% (4.22)	4% (2.84)	0.21	1% (1.18)	* − 0.33*	* − 0.52*	3% (2.37)	0.02	0.33
Harry	3% (3.58)	8% (4.09)	**0.71**	7% (2.54)	**0.74**	* − 0.07*	11% (5.72)	**0.79**	*0.24
Dominic	0% (0.00)	3% (4.83)	0.50	0% (0.00)	0.00	* − 0.50*	0% (0.00)	0.00	0.00
Object/gesture imitation									
Charles	8% (3.85)	9% (3.44)	0.11	12% (7.88)	0.44	0.26	8% (6.36)	* − 0.07*	* − 0.44
Muhammed	5% (4.41)	1% (2.44)	* − 0.48*	7% (3.33)	0.22	***0.86***	7% (5.00)	0.19	* − 0.04
Zack	1% (1.92)	5% (2.82)	**0.71**	5% (4.13)	**0.67**	* − 0.09*	7% (4.75)	***0.89***	*0.19
Elijah	2% (2.55)	4% (5.24)	0.11	5% (4.38)	0.50	0.21	10% (3.04)	***1.00***	0.56
Steve	1% (1.64)	5% (3.93)	**0.76**	4% (4.08)	0.40	* − 0.32*	6% (2.37)	***0.87***	0.40
Harry	2% (1.58)	8% (6.91)	0.57	5% (3.54)	0.49	* − 0.27*	6% (3.40)	**0.78**	0.18
Dominic	6% (5.32)	8% (5.35)	* − 0.07	13% (6.06)	*0.40	0.53	10% (4.94)	*0.23	*-0.28*

*indicates that baseline trend was corrected for. Bold indicates large positive effect sizes (> 0.50); bold-italics indicates very large positive effect sizes (> 0.80); italics indicates a negative effect size (< 0)

#### Joint Engagement

**Fig. 2:**
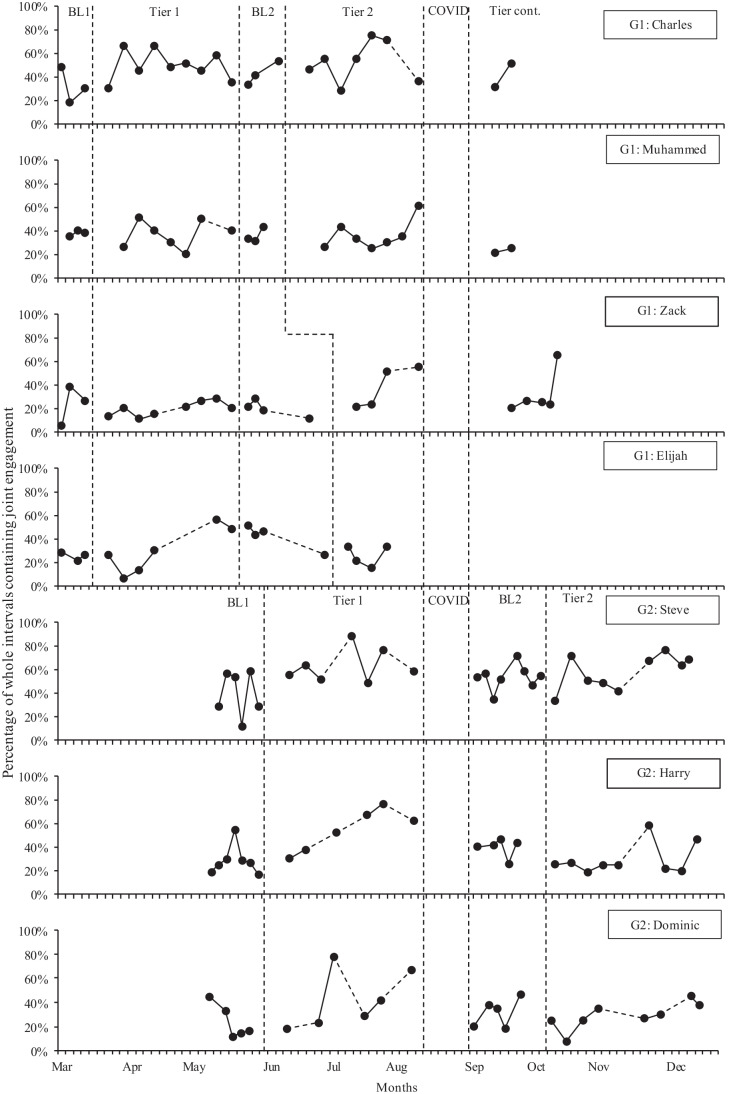
Percentage of child engagement across individual children and study phases. Note: BL, Baseline; COVID, COVID lockdown; Tier 2 cont., Tier 2 continued after the COVID lockdown. G1, Group 1; G2, Group 2. A dashed line between data points indicates a gap of more than 1 week

Figure 2 shows the percentage of whole intervals in which each child demonstrated joint engagement with their parent across all phases. There was an increase in joint engagement levels between Baseline 1 and Tier 1 for five children as shown by increased mean joint engagement, and small (Elijah), moderate (Dominic), large (Charles and Steve), and very large (Harry) positive Tau-U effect sizes. Generally, these gains were maintained through Baseline 2 but with a decreasing trend for Elijah. Muhammed and Zack had stable trends and no large changes in their mean engagement through the first three phases. Although Muhammed had an increasing trend in Tier 2 pre-COVID, overall, there was a negative Tau-U effect size between Muhammed’s Baseline 1/Baseline 2 and Tier 2. Harry and Elijah’s engagement levels also decreased during Tier 2 to return to similar levels as pre-intervention. However, between Baseline 2 and Tier 2, Zack’s mean joint engagement increased with a large positive Tau-U effect size. Dominic, Steve, and Charles also had higher mean joint engagement in Tier 2 than in Baseline 2, with high variability for Charles, an increasing trend for Steve, and a relatively flat trend for Dominic. Tau-U effect sizes show small (Zack, Dominic), moderate (Steve), and large (Charles) improvements in joint engagement between Baseline 1 and Tier 2 for some children, small (Harry) to moderate (Muhammed) decreases for others, and very little change for one (Elijah). Across all children, joint engagement levels were lower immediately following the COVID lockdown than they were pre-lockdown.

#### Functional Utterances

**Fig. 3:**
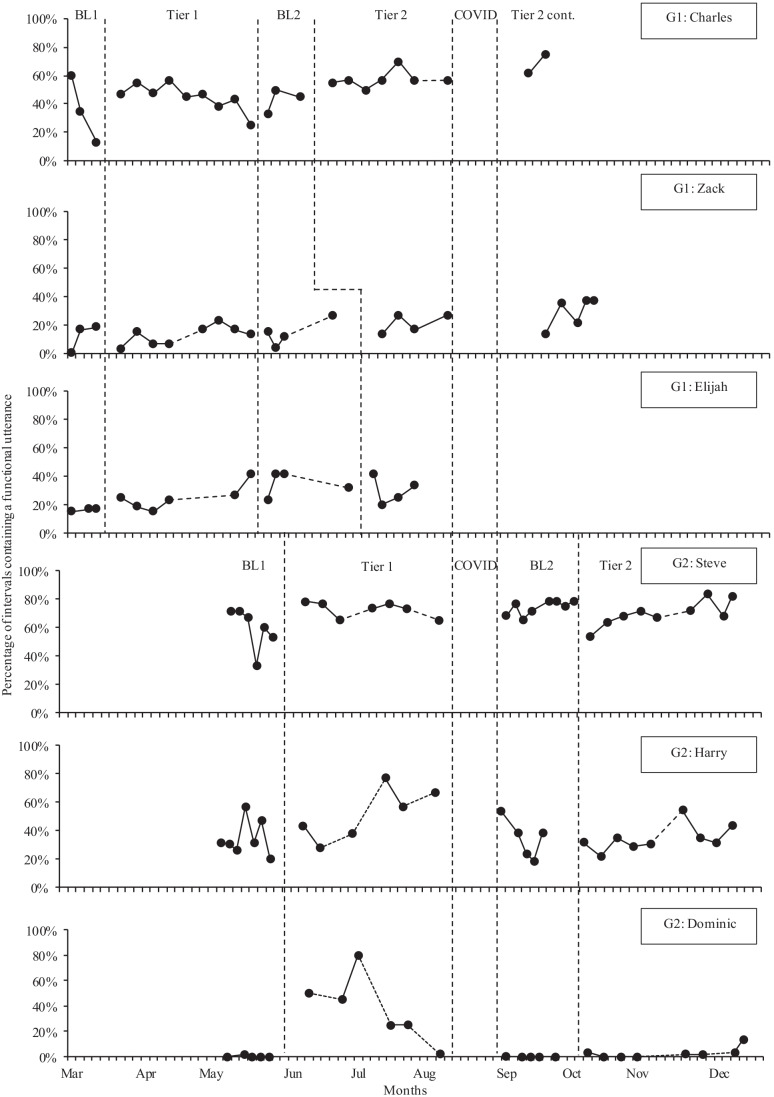
Percentage of child functional utterances across individual children and study phases. Note: BL, Baseline; COVID, COVID lockdown; Tier 2 cont., Tier 2 continued after the COVID lockdown. G1, Group 1; G2, Group 2. A dashed line between data points indicates a gap of more than 1 week

**Fig. 4:**
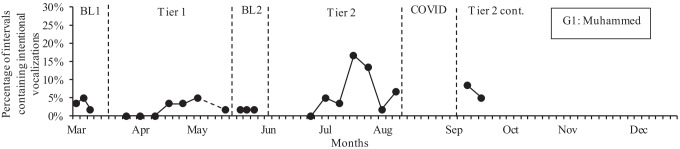
Percentage of intentional vocalizations for Muhammed across study phases. Note: BL, Baseline; COVID, COVID lockdown; Tier 2 cont., Tier 2 continued after the COVID lockdown. G1, Group 1; G2, Group 2. A dashed line between data points indicates a gap of more than 1 week

Figure 3 shows the percentage of intervals containing functional utterances for Charles, Zack, Elijah, Steve, Harry, and Dominic across all phases. For Muhammed, functional utterances were not a developmentally appropriate outcome. Instead, Muhammed’s intentional vocalizations are reported separately in Fig. 4. Charles showed a sharp decreasing trend in functional utterances during baseline, then increased his mean functional utterances during Tier 1 (albeit with a decreasing trend), maintained mean functional utterances at a similar level during Baseline 2, then further increased his mean functional utterances in Tier 2 with an increasing trend. Tau-U effect sizes for Charles’ functional utterances showed small to very large improvements between each phase. Zack showed little change in mean functional utterances across Baseline 1, Tier 1, and Baseline 2. Then, had an increasing trend and higher mean functional utterances in Tier 2. Tau-U scores for Zack’s functional utterances were only positive at Tier 2, with a moderate effect size between Baseline 1 to Tier 2, and Baseline 2 to Tier 2. Elijah had a stable trend in Baseline 1 and an increasing trend in functional utterances through Tier 1 and Baseline 2, with higher mean utterances in each subsequent phase. However, mean utterances decreased in Tier 2, with a decreasing trend. Tau-U scores for Elijah’s functional utterances showed large to very large positive effect sizes across all phases except Baseline 2 to Tier 2 when there was a small decreasing effect size. After a decreasing trend in Baseline 1, Steve’s functional utterances increased immediately at the beginning of Tier 1. Mean functional utterances were higher in all phases than in Baseline 1, though there was a slight decrease in mean functional utterances between Baseline 2 and Tier 2. Overall, Tau-U scores show moderate to large positive effect sizes between Baseline 1 and all other phases, but with a moderate decrease between Baseline 2 and Tier 2. Harry’s mean functional utterances improved between Baseline and Tier 1 with a strong increasing trend in Tier 1. There was a decreasing trend during Baseline 2, and mean functional utterances returned to Baseline 1 levels for both Baseline 2 and Tier 2. Tau-U effect sizes for Harry’s functional utterances mirror the descriptive statistics with a moderate positive effect size between Baseline 1 and Tier 1 only. Dominic spoke very rarely during Baseline 1. He immediately increased his functional utterances in Tier 1, with a particularly high level of functional utterances in his third session, but with a decreasing trend thereafter. Dominic did not use any functional utterances in Baseline 2, but then increased his functional utterances during Tier 2. Tau-U scores show positive effect sizes between Baseline 1 and Tier 1/Tier 2, as well as between Baseline 2 and Tier 2.

#### Intentional Vocalizations

Figure 4 shows the percentage of intervals containing intentional vocalizations for Muhammed across all phases. Muhammed’s rates of intentional vocalizations were very low (mean: 3%) in Baseline 1. This further decreased to a mean of 2% across both Tier 1 and Baseline 2. However, in Tier 2, Muhammed’s intentional vocalizations increased to a mean of 7%, with a moderate positive effect size between Baseline 1 and Tier 2 (Tau-U = 0.41) and a large positive effect size between Baseline 2 and Tier 2 (Tau-U = 0.67).

#### Imitation

**Fig. 5:**
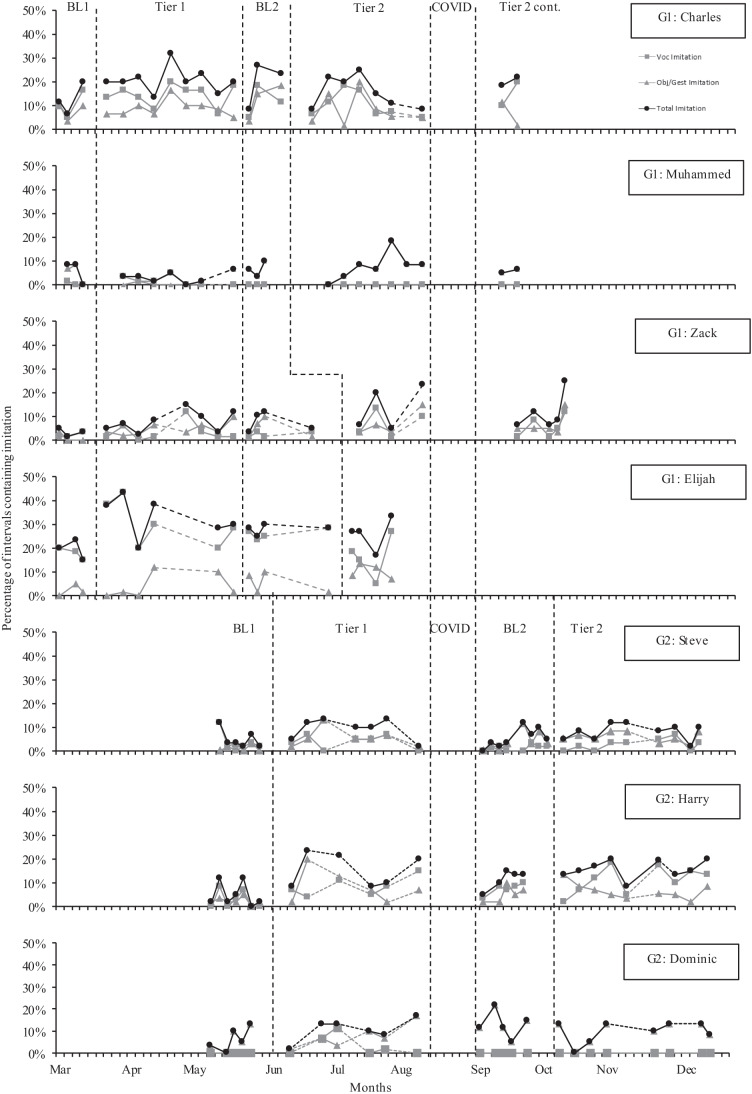
Percentage of child imitation across children and study phases. Note: BL, Baseline; COVID, COVID lockdown; Tier 2 cont., Tier 2 continued after the COVID lockdown. G1, Group 1; G2, Group 2. A dashed line between data points indicates a gap of more than 1 week

Figure 5 shows the percentage of intervals containing both vocal imitation and object/gesture imitation for each child across all phases. Vocal imitation and object/gesture imitation are described separately.

##### Vocal Imitation

Vocal imitation was generally very low for Muhammed, Steve, and Dominic across all phases and did not show any large increases or decreases in trend, means, or effect sizes. Charles had higher levels of vocal imitation but also did not show any large changes in trend, means, or effect sizes across phases. Elijah had the highest vocal imitation in baseline and increased his mean vocal imitation across Tier 1 and Baseline 2 above Baseline 1 levels (with very large positive Tau-U effect sizes), before decreasing below Baseline 1 levels in Tier 2 (a small negative Tau-U effect size between Baseline 1 and Tier 2). Harry and Zack generally increased their vocal imitation across phases with moderate (Zack) or large (Harry) positive Tau-U effect sizes for vocal imitation between Baseline 1 and Tier 2.

##### Object/Gesture Imitation

Object/gesture imitation rates were low across all children in Baseline 1. There were mixed increases and decreases across Tier 1. However, in Baseline 2, all children had increased their mean rate of imitation above Baseline 1 levels with moderate to large Tau-U effect sizes. Charles did not maintain this across Tier 2 and returned to Baseline 1 mean levels of object/gesture imitation in Tier 2. Muhammed and Dominic did not make any additional increases in Tier 2 but maintained higher mean levels of imitation than in Baseline 1. In Tier 2, Zack, Elijah, Steve, and Harry made additional increases in mean object/gesture imitation above Baseline 2, to show large or very large Tau-U effect sizes between Baseline 1 and Tier 2.

### Relation Between Parent Fidelity and Child Outcomes

Table 7 shows the relation between parent fidelity and each child outcome based on Spearman’s correlation coefficient across all parent–child dyads.

**Table 7:** Spearman’s correlation coefficient (*R*_s_) for the relationship between parent fidelity and child outcomes across all participants

Parent fidelity	Engagement (*p*-value)	Functional Utterances/intentional vocalizations (*p*-value)	Vocal imitation (*p*-value)	Object/gesture Imitation (*p*-value)
Holly	.70**, (p < *.001)*	.15, (*p* = .497)	.05, (*p* = *.*836)	.30, (*p* = .160)
Kiran	.43*, (***p* = .049)**	.08, (*p* = .712).^IV^	− .45*, (*p* = .035)	.35, (*p* = .110)
Heather	.49*, (*p* = .016)	.49*, (*p* = .**016)**	.45*, (*p* = .028)	.63**, (p = *.001)*
Kelly	.86**, (p < *.001)*	.69**, (p = *.002)*	.42, (*p* = .096)	.38, (*p* = .130)
Amanda	.68**, (p < *.001)*	.66**, (p < *.001)*	.32, (*p* = .084)	.32, (*p* = .083)
Merry	.69**, (p < *.001)*	.63**, (p < *.001)*	.22, (*p* = .28)	.09, (*p* = .656)
Sam	.79**, (p < *.001)*	.42*, (*p* = .042)	.34, (*p* = .108)	.37, (*p* = .076)

Bold indicates a moderate relationship, bold-italics indicates a strong relationship. Italics indicates a negative relationship. ^IV^Intentional vocalizations, when functional utterances were not a developmentally appropriate outcome measure

^*^Correlation is significant at the 0.05 level (2-tailed); **correlation is significant at the 0.01 level (2-tailed)

For five parents (Holly, Kelly, Amanda, Merry, and Sam) there was a significant strong positive relationship between parent fidelity and child engagement. For the other two parents (Kiran and Heather), there was a significant moderate positive relationship between parent fidelity and child engagement. There were significant strong positive relationships between parent fidelity and child functional utterances for three parents (Kelly, Amanda, and Merry) and significant moderate positive relationships for two parents (Heather and Sam). There was no significant association between parent fidelity and child functional utterances for two parents (Holly and Kiran). For Kiran, there was a moderate negative relationship between fidelity and vocal imitation. For Heather, there was a moderate positive relationship between fidelity and vocal imitation, and a strong positive relationship between fidelity and object/gesture imitation. Across all other measures, there were no significant relationships.

### Intervention Hours

Supplementary Table 4 shows the weekly time each parent reported that they, or others in their household, spent using the ESDM strategies with their child across Tiers 1 and 2. This shows the reported child intervention hours. The percentage of weekly logs that were completed and returned is also shown. The percent of coaching sessions that each parent attended is reported across Tiers 1 and 2. This shows the parent intervention hours.

In both Tier 1 and Tier 2, reported child intervention hours varied significantly across families, and across weeks for individual families. However, all families found some time to use the strategies with their child each week (except Heather who reported 0 h for 1 week in Tier 2 due to sickness). The mean time parents spent using ESDM strategies with their child per week was 6 h 32 min per week in Tier 1, and 6 h 20 min in Tier 2. Sam reported the lowest mean intervention hours; 1 h 46 min in Tier 1, and 1 h 16 min in Tier 2. Amanda reported the highest mean intervention hours; 11 h 30 min in Tier 1, and 11 h 22 min in Tier 2. In Tier 1 there was a high return rate of the weekly logs, with all parents returning more than 89% of the logs except Kiran (44% returned).

Holly, Kiran, and Merry increased the mean number of hours they reported using ESDM strategies with their child each week in Tier 2 compared to Tier 1. Amanda and Sam continued to report a similar mean number of hours spent using ESDM strategies with their child in Tier 2 as they had in Tier 1. Heather decreased the mean number of hours she reported using ESDM strategies with her child in Tier 2; however, she only returned logs for the first four weeks of Tier 2.

### Procedural Integrity and Interobserver Agreement

There was high procedural integrity across 20% of the sessions in each phase of the intervention that were evaluated. Across Baseline 1 and Baseline 2, there was a mean of 95% procedural integrity (*n* = 11, range: 80–100%). Procedural integrity for the group coaching sessions was 98% (*n* = 4, range 92–100%). Procedural integrity (coaching fidelity) during the 1:1 coaching sessions was 100% across all sessions (*n* = 13).

There was adequate mean interobserver agreement (78–97%) across all parent and child outcomes in the 20% of data that was coded for agreement (see supplementary Table 5).

## Discussion

There is a growing need for effective and efficient supports for young autistic children. This study explored the effectiveness of a low-intensity two-tiered approach to coaching parents in the P-ESDM. Parents initially participated with their children in a 9- or 10-week group coaching program, then in 10 sessions of 1:1 parent coaching. All parents improved in their use of ESDM strategies during the group coaching program. The addition of 1–1 coaching was helpful for further increasing the ESDM fidelity of some, but not all parents. Parenting stress levels were lower post-intervention than pre-intervention for most parents, with no parents showing high or clinically significant levels of stress post-intervention. The child results show that all children improved above baseline levels across at least two domains. Specifically, four children showed improvement in engagement, six children improved in their functional utterances or intentional vocalizations, two children improved their vocal imitation, and six children improved their object and gesture imitation.

For three parents, Holly, Kelly, and Amanda, 9–10 weeks of group P-ESDM appeared to be sufficient to support them to use over 75% of ESDM strategies accurately either during the coaching phase or in the following reinstated baseline conditions. Adding an additional 10 weeks of 1:1 coaching helped some parents to make further small improvements to their overall ESDM fidelity, and all parents except Sam demonstrated above 75% fidelity in at least one session by the end of the 1:1 coaching. However, Kelly and Merry both showed decreases in overall fidelity during the 1:1 coaching phase, suggesting that the addition of 1:1 coaching was not helpful for all parents. This is similar to other research that has found that some parents do not respond to 1:1 P-ESDM (Waddington et al., 2019).

It is also important to note that there was variability with respect to the parents’ use of ESDM strategies across all phases in this intervention. For example, none of the parents maintained a high level of fidelity over all sessions. This may be because the comprehensive nature of the ESDM intervention may have made it more difficult to maintain fidelity across the wide range of activities and target skills associated with the program (Wainer et al., 2021). The 3-week COVID lockdown that occurred in New Zealand also likely impacted the parents’ maintenance of fidelity with respect to implementing ESDM strategies. Indeed, fidelity levels dropped immediately following the lockdown period.

In addition to variable maintenance of fidelity levels, not all parents reached 80% fidelity. Sam’s overall fidelity relatively remained low for example, and he had no sessions where fidelity was above 75%. Other P-ESDM research has also found that not all parents reach high levels of fidelity (Rogers et al., 2012b; Vismara et al., 2009, 2013, 2016; Waddington et al., 2019). There are some similarities between Sam, Heather, and Kiran who had the lowest overall levels of fidelity. It is possible that the coaching was less effective for these parents for several reasons. First, there may have been language barriers to understanding the strategies for Sam and Kiran who spoke English as a second language. Second, work and family time constraints may have made it difficult to practice the ESDM at home for Kiran, Heather, and Sam who all reported low intervention hours. For example, Sam worked full-time and was a father of five children. Third, Sam and Heather had some indication of Broad Autism Phenotype (BAP) profiles. A high BAP score can be associated with difficulties “learning new strategies, organizing and planning structured practices with children, and generalizing skills to new situations” (Shalev et al., 2020, p. 2). Fourth, Sam and Heather had high-stress levels on the PSI at intake, and stress may negatively impact parent engagement and learning in PMIs (Shalev et al., 2020). These similarities across parents suggest that these factors warrant further investigation as potential barriers to parent learning of the ESDM and highlight the need for programs that better meet the needs of these parents. For example, for parents who do not have much time to implement ESDM at home, perhaps a therapist implemented ESDM would be a better fit. For parents with high-stress levels, modified ESDM approaches that include stress-reduction strategies (e.g., Weitlauf et al., 2020) may not only reduce parent stress levels but also help those parents to learn to use the ESDM with higher fidelity.

In this study, parenting stress was reduced for five out of six parents who completed the post-assessment measures. Pre-intervention, four parents reported some high or clinically significant levels of stress. However, post-intervention, no parents had high or clinically significant levels of stress. This aligns with previous research that has demonstrated that participating in P-ESDM can reduce parenting stress (Rogers et al., 2012b; Weitlauf et al., 2020; Zhou et al., 2018). Reducing parental stress is a clinically significant outcome, as parents of autistic children report much higher levels of stress than parents of typically developing children and children with other disabilities (Hayes & Watson, 2013). This impacts negatively on parents and is associated with poorer outcomes for their children (Crowell et al., 2019; Shalev et al., 2020).

Parent implemented ESDM has been shown to positively impact child outcomes (Vismara et al., 2009, 2012, 2013; Mirenda et al., 2022; Waddington et al., 2021; Zhou et al., 2018). In this study, there were moderate to strong positive relations between parent fidelity and child engagement across all parent–child dyads and moderate to strong positive associations between parent fidelity and child functional utterances across the six dyads for whom this was a relevant outcome measure. This suggests that the changes in child engagement and functional utterances that were observed in this study were possibly due to the parents learning to implement the ESDM strategies (Wainer & Ingersoll, 2013). Positive associations between parent fidelity and child outcomes have been found in three other studies (Rogers et al., 2019; Vismara et al., 2013; Waddington et al., 2020). Unlike the findings from Rogers et al. (2019), in this study, there does not seem to be a threshold effect of parent fidelity improvement on child outcomes, but rather, all parents’ increases in accurate strategy use were associated with increases in engagement and functional utterances, even when those parent improvements were well below fidelity.

Finding a relation between ESDM strategy use and child engagement would seem to have some clinical importance given that joint engagement is a reported area of difficulty for many autistic children (Kasari et al., 2010). In addition, lower engagement levels may reduce the number and quality of social learning opportunities that occur across a child’s day (Dawson et al., 2012). Increased joint engagement can have positive flow-on effects for child language (Shih et al., 2021), and be a mediator of the effects of early intervention on cognition, language, and adaptive behavior outcomes (Dawson et al., 2012).

Similarly, even small improvements in child functional communication could be seen as a promising outcome given that communication is a core challenge associated with autism (Tiede & Walton, 2019). All the children in the present study had communication delays as assessed by the Vineland-3. Better childhood language skills may be associated with improved outcomes for autistic adolescents and adults, including reduced autism characteristics, higher adaptive behavior scores, and better social outcomes (Magiati et al., 2014).

For imitation, there were only small positive associations between fidelity and imitation for most parents on both measures of child imitation. One explanation for this is that only spontaneous instances of imitation were recorded. Given that levels of spontaneous imitation were generally very low in the baseline for these children and imitation is one of the skills most impacted in autistic children, it is possible that parents needed to prompt their children to imitate much of the time. It is also possible that creating opportunities to imitate was a difficult skill for parents to learn, as it requires parents to initiate learning opportunities rather than respond to child behaviors which are harder strategies to implement in play (Stahmer et al., 2017; Waddington et al., 2020). Despite the small improvements in object/gesture imitation across all children, and in vocal imitation for some children, overall rates of spontaneous imitation were very low across all phases. Future PMI research could consider measuring prompted behaviors in addition to spontaneous behaviors to help to understand how often parents are targeting imitation with their children, and whether there is any relationship between prompted imitation opportunities and child spontaneous imitations.

The group coaching approach used in Tier 1 of this research could be seen as somewhat novel within the ESDM literature. It was very low intensity (with only two to three clinician hours across seven participating families per week), over a shorter timespan than any other P-ESDM literature (maximum of 10 weeks). Additionally, most families missed at least one session of the group coaching program, therefore further reducing the parent intervention hours. Yet, this very low-intensity program was effective in improving many parent and child outcomes. This has significant clinical implications as group delivery of parent coaching interventions is a very efficient and cost-effective service delivery approach (Minjarez et al., 2011). However, from a stepped care approach, it is possible that some parents would benefit from an even less intensive first tier of intervention. Wainer et al. (2021) found that some parents achieved fidelity in reciprocal imitation training (a focused NDBI) through the completion of self-paced online modules only. Future P-ESDM studies could evaluate the impact of parents completing the online ESDM modules at Help is in Your Hands without direct coaching as a first tier. Additionally, future research could investigate the impact of a group P-ESDM format with a longer duration. Not all parents demonstrated the use of the ESDM at fidelity after the 9- to 10-week group program in this study. However, some parents were able to achieve fidelity in later tiers of intervention. It is not clear whether these improvements were due to having more time to learn the strategies, or due to the nature of 1:1 coaching support. Exploring options for higher intensity tiers, with more individualized supports for parents who do not respond to lower tiers is also a key priority for future research.

A strength of this research was the inclusion of parent reporting of intervention hours during the days between coaching sessions. Child intervention hours or treatment dose is rarely included in PMI literature (Nevill et al., 2018; Wainer et al., 2021). It was promising to see that all families found some time during their weeks to practice intervention strategies with their children as this suggests that the ESDM strategies were generalizable outside of the clinic setting. However, on average, parents in this study reported using the ESDM strategies with their child less than 7 h per week. Given that autistic children in New Zealand receive an average of only 2 h per week of intervention (Kasilingam et al., 2021), children in this study were highly unlikely to be accessing the recommended 20 + hours per week of EI (National Research Council, 2001). Therefore, parents may be a “promising alternative source of early intervention” in theory (Nevill et al., 2018, p. 84); however, in practice, requiring parents to be the sole source of EI for their own child may be unrealistic.

### Limitations and Future Research

There are several limitations to this present study. First, the small sample size and a lack of comparison or control group mean that the findings of this study are limited to the seven parent–child dyads and need to be replicated with a larger and more diverse sample of participants. Second, the first author was the parent coach as well as the primary rater of parent and child outcomes and therefore was not blind to the treatment phase which may have biased the results. Third, the parent coach was a highly qualified doctoral student with multiple years’ experience with the ESDM, this may mean that the results of this study have limited replicability in community contexts. Fourth, only five of the seven primary participants returned their PSI forms post-intervention. Fifth, one family withdrew from this research mid-way through Tier 2 and declined to complete any post-assessment forms or interviews; therefore, important data about the effectiveness and acceptability of this intervention may be missing. Sixth, this study did not evaluate child outcomes on any standardized measures. Child change on standardized developmental measures is the most rigorous evidence of child improvement and should be included in future research of this kind. There is also limited understanding of how much change in child outcomes is required for the improvements to be clinically significant. Seventh, there was no measure of how well parents maintained the use of these strategies post-intervention. Even over a 3-week lockdown, all parents reduced their fidelity; therefore, maintenance of skills over time is a concern for this group.

In addition to the areas of future research already mentioned, understanding barriers to parent learning of the ESDM also requires further investigation. Additionally, given the promising results from the group parent coaching intervention, future randomized and controlled studies of a group approach to P-ESDM are warranted.

## Supplementary Information

Below is the link to the electronic supplementary material.

Supplementary file1 (DOCX 54 KB)

## Declarations

### Data Repository

Data pertaining to this project can be accessed at https://doi.org/10.17605/OSF.IO/JTV96.
