# Supplementary material for: Evaluating a Two-Tiered Parent Coaching Intervention for Young Autistic Children Using the Early Start Denver Model
Source: Adv Neurodev Disord. 2022 May 30;6(4):473–93. doi: 10.1007/s41252-022-00264-8 (PMC9149339; doi:10.1007/s41252-022-00264-8)
Supplement: Supplementary file 1 — Supplementary file1 (DOCX 54 KB) [file 41252_2022_264_MOESM1_ESM.docx]

| **Supplementary Table 1**  *Procedural Integrity, Tier 1 Coaching Sessions.* |  | | |
| --- | --- | --- | --- |
| **Date:__________________________________** | **Observer:______________________________** | | |
| Fidelity Item | | Yes / No | Notes/Comments: |
| Arrival / set up | |  |  |
| 1. Children and parents were greeted at the beginning of playgroup | | Yes / No |  |
| 1. Children were offered toys and/or activities by playgroup assistants | | Yes / No |  |
| 1. Parents and the playgroup facilitator (therapist) had an adult sized table and chairs to sit at to discuss the weeks’ content | | Yes / No |  |
| Content | |  |  |
| 1. The playgroup facilitator checked in with the parents about their use of strategies/goals from the previous week | | Yes / No |  |
| 1. The playgroup facilitator presented one new parent coaching topic | | Yes / No |  |
| 1. Each parent had a written handout pertaining to the topic of the week | | Yes / No |  |
| 1. Parents had the opportunity to ask questions of the playgroup facilitator about the topic | | Yes / No |  |
| Practice | |  |  |
| 1. At least 10 minutes of the session was allocated for parents to practice the week’s topic with their own child (parents all practiced at the same time). | | Yes / No |  |
| 1. The playgroup facilitator observed and/or provided live coaching to each parent during the parents’ interactions with their children | | Yes / No |  |
| Reflection and goal setting | |  |  |
| 1. Parents and the playgroup facilitator reflected collaboratively on the practice time | | Yes / No |  |
| 1. Each parent had the opportunity to set a goal for the coming week | | Yes / No |  |
| Duration | |  |  |
| 1. The playgroup session lasted between 60-90 minutes in total | | Yes / No |  |

| **Supplementary Table 2**  *Procedural Integrity, Baseline Videos.* | | |  | |
| --- | --- | --- | --- | --- |
| **Session Code: __________________________** | | | **Observer:______________________________** | |
|  | **Fidelity Item** | | **Score:**  **Yes (1) No (0)** | |
| 1 | Session occurred in a clinic room that contained a child-sized table and chair, floor space to play, and a range of appropriate toys.  OR session occurred at the family’s home. | |  | |
| 2 | The video recording lasted 10-minutes, OR the video lasted longer than 7-minutes and was stopped on parent request. | |  | |
| 3 | Interactions were only between the primary parent and child participants. If there were other adults or children present, they did not interact with or otherwise disrupt interactions between the primary parent and child. | |  | |
| 4 | The researcher or research assistant did not interact with the parent or child during the video. | |  | |
| 5 | The parent engaged with or attempted to engage with the child during the video. | |  | |

| **Supplementary Table 3**  *Child Dependent Variables and Definitions* | |  |
| --- | --- | --- |
| Target Behaviour | Definition | Measurement |
| Joint Engagement | The child was “jointly engaged” for any *whole interval* in which the child was actively participating with their parent. This included:  (a) orienting their face towards their parent;  (b) watching the adult’s actions with an object;  (c) giving or taking objects from an adult;  (d) using any form of communication that is clearly directed towards the parent;  (e) following an adult’s instructions;  (f) imitating the adult; or  (g) responding to the parent’s actions in a way that continues the joint activity (e.g. running away from the parent in a chase game). | Whole-interval recording  (10-s intervals) |
| Imitation | *Any instance* in which the child spontaneously copied a sound (vocal imitation) or an action (object or gestural imitation) modelled by the parent, within 10-seconds of the parent-model, without any prompting from the parent. Does not need to be accompanied by eye contact.  *Vocal Imitation:*  A spontaneous imitation that is a phonetically correct approximation of any word, phrase, or vocal sound made by the parent.  *Object or Gesture Imitation:*  A spontaneous imitation of any adult action on an object, or any gesture that the adult makes. | Partial-interval recording  (10-s intervals) |
| Functional Utterance | *Any instance* of the child directing spoken language to the parent. The utterance does not need to be accompanied by eye contact, but must be clearly directed to the adult. (e.g. answering an adult’s question, labelling an adult’s action, paired with a gesture aimed at the adult, speaking and then waiting for an adult’s response, or looking at the parent before/after the utterance). It must also be:   1. spontaneous (not prompted or modelled by the parent within 10 seconds of the child’s utterance); 2. contextually related to the interaction or task (e.g. not unrelated speech, not repetitions of the child’s own speech); 3. a phonetically correct approximation of the correct word, sound effect, or word combination (e.g. not saying horse for a cow). | Partial-interval recording  (10-s intervals) |
| Intentional Vocalisations | Intentional vocalisations were only coded when functional utterances were not a developmentally appropriate measure for the child. An intentional vocalisation is an intentional sound that is not a phonetically correct approximation of the correct word or phrase.  Intentional vocalisations were coded as *any instance* of the child spontaneously directing an intentional vocal sound to the parent. Generic sounds such as crying, whining, humming, or laughing were not counted. | Partial-interval recording  (10-s intervals) |

| **Supplementary Table 4**  *Treatment Dosage: Reported Time Spent Using ESDM Strategies with Child Per Week, and Percentage of Coaching Sessions Attended.* | | | | | | | | |
| --- | --- | --- | --- | --- | --- | --- | --- | --- |
|  | Tier 1 | | |  | Tier 2 | | | |
| Parent | Mean reported time using strategies per week: hh:mm (range) | % of weekly logs returned | % of Tier 1 sessions attended |  | Mean reported time using strategies per week: hh:mm (range) | Increase/ decrease in mean time from Tier 1 | % of weekly logs returned | % of Tier 2 sessions attended |
|  |  |  |  |  |  |  |  |  |
| Holly | 06:15 (03:45 – 09:20) | 100% | 100% |  | 10:05 (04:15 – 18:00) | +03:50 | 67% | 100% |
| Heather | 10:40 (07:15 – 21:00) | 89% | 90% |  | 03:27 (00:00 – 08:00) | -07:13 | 44% | 100% |
| Kiran | 02:54 (00:37 – 09:30) | 44% | 80% |  | 04:09 (00:36 – 12:40) | +01:15 | 67% | 100% |
| Kelly | 08:27 (04:55 – 15:45) | 100% | 70% |  | NR |  | 0% | 50% |
| Amanda | 11:30 (06:00 – 14:30) | 89% | 100% |  | 11:22 (03:30 – 19:00) | -00:08 | 90% | 100% |
| Merry | 04:15 (01:18 – 09:09) | 100% | 78% |  | 07:44 (07:10 – 08:55) | +03:29 | 100% | 100% |
| Sam | 01:46 (01:01 – 02:53) | 89% | 67% |  | 01:16 (01:09 – 01:45) | -00:30 | 90% | 100% |
| Mean | 06:32 (00:37 -09:30) |  |  |  | 6:20 (00:00 – 19:00) |  |  |  |
| *Note:* hh:mm = hours:minutes. NR = not reported. | | | | | | | | |

| **Supplementary Table 5**  *Interobserver Agreement on Parent and Child Dependent Variables* | | | | |
| --- | --- | --- | --- | --- |
| Dependent Variables | % adjacent agreement  (% exact agreement) | Range adjacent agreement (range exact agreement) | % of interval agreement | Range of interval agreement |
| Parent |  |  |  |  |
| Fidelity | 97%  (61%) | 62%-100%  (23%-92%) | - | - |
| Child |  |  |  |  |
| Joint engagement | - | - | 78% | 43%-97% |
| Functional Utterances | - | - | 89% | 72%-100% |
| Intentional Vocalizations | - | - | 90% | 77%-97% |
| Vocal Imitation | - | - | 96% | 87%-100% |
| Object/Gesture Imitation | - | - | 94% | 82%-100% |
